# Validation and Comparison of Pediatric Appendicitis Scores and Management Strategies (Project SPASMS): Protocol for a Prospective Multicenter Observational Study

**DOI:** 10.2196/67941

**Published:** 2025-06-27

**Authors:** Wei Hao Lee, Sharon O'Brien, Elizabeth J Mckinnon, Simon Craig, Stuart Dalziel, Michael Collin, Natalie Phillips, Franz E Babl, Sarah Julia Davidson, Shane George, Shefali Jani, Doris Tham, Viet Tran, Meredith L Borland

**Affiliations:** 1 Division of Paediatrics School of Medicine University of Western Australia Crawley Australia; 2 Emergency Department Perth Children's Hospital Nedlands Australia; 3 Division of Emergency Medicine School of Medicine University of Western Australia Nedlands Australia; 4 Institute for Paediatric Perioperative Excellence The University of Western Australia Perth Australia; 5 Biostatistics The Kids Research Institute Australia Nedlands Australia; 6 Paediatric Emergency Department Monash Medical Centre Monash Health Clayton Australia; 7 Department of Paediatrics School of Clinical Sciences Monash University Clayton Australia; 8 Emergency Department Starship Children's Health Auckland New Zealand; 9 Department of Surgery and Paediatrics Child and Youth Health University of Auckland Auckland New Zealand; 10 Department of Surgery Perth Children's Hospital Nedlands Australia; 11 Emergency Department Queensland Children's Hospital Brisbane Australia; 12 Child Health Research Centre University of Queensland South Brisbane Australia; 13 Departments of Paediatrics and Critical Care University of Melbourne Parkville Australia; 14 Emergency Department Royal Children's Hospital Parkville Australia; 15 Emergency Research Group Murdoch Children's Research Institute Parkville Australia; 16 Emergency Department Sunshine Coast University Hospital Sunshine Coast Australia; 17 Children's Emergency and Critical Care Collaborative Research Group Gold Coast University Hospital Gold Coast Australia; 18 Menzies Health Institute Queensland Griffith University Gold Coast Australia; 19 Faculty of Medicine University of Queensland Brisbane Australia; 20 Department of Emergency Medicine Children's Hospital at Westmead Sydney Australia; 21 Faculty of Medicine and Health University of Sydney Sydney Australia; 22 Sunshine Paediatric Emergency Department Western Health Melbourne Australia; 23 Royal Hobart Hospital Tasmanian Health Service Hobart Australia; 24 Menzies Institute for Medical Research Tasmanian School of Medicine University of Tasmania Hobart Australia

**Keywords:** pediatric, emergency medicine, clinical prediction scores, appendicitis, abdominal pain

## Abstract

**Background:**

Abdominal pain is a common reason for children to attend the Emergency Department (ED) with acute appendicitis being the most common surgical cause. Various clinical prediction scores (CPSs) have been developed to assist in determining the risk of appendicitis; however, CPSs have been inadequately validated in children and haphazardly adopted in Australia and New Zealand (ANZ) EDs.

**Objective:**

This study aims to compare and validate various CPSs for diagnosing pediatric appendicitis in children presenting to ANZ EDs.

**Methods:**

This prospective multicenter observational study across 10 ANZ EDs is recruiting children 5-17 years presenting to participating EDs with acute right-sided or generalized abdominal pain lasting ≤7 days and clinician suspicion of appendicitis. CPSs will be calculated by the study team from clinician-recorded data and clinician gestalt recorded on a visual analog scale. Accuracy of CPSs will be assessed by the area under the receiver operating characteristic curve and proportions correctly identified as either low-risk or high-risk based on the CPSs published cutoffs. The final diagnosis of appendicitis will be confirmed on histopathology, and the absence of appendicitis confirmed by telephone, email, or a combination of both to follow up for those discharged directly from ED.

**Results:**

This study received funding in July 2023 and started enrolment in August 2023. As of October 2024, we have enrolled and completed follow-up on 1227 participants with an expected end date in mid-2025.

**Conclusions:**

This study aims to determine the best-performing CPS for diagnosing pediatric appendicitis in ANZ EDs. Implementation of this CPS in ANZ EDs has the potential to reduce health care costs, rationalize the use of health care resources, and improve the management and outcomes of childhood appendicitis.

**Trial Registration:**

Australian New Zealand Clinical Trials Registry ACTRN12622001293752; https://tinyurl.com/242wwenx

**International Registered Report Identifier (IRRID):**

DERR1-10.2196/67941

## Introduction

### Background

Abdominal pain is a common reason for children to attend the emergency department (ED), with acute appendicitis being the most frequent cause requiring surgical intervention [1]. Many clinical prediction scores (CPSs) [2-31] have been developed and ED clinicians are encouraged to use CPSs to assist with determining the risk of appendicitis [32]. Most CPSs involve calculating a score based on combinations of clinical features and laboratory findings to classify patients into low-, intermediate-, or high-risk. The most frequently used CPSs in children are the Alvarado score [2], the Pediatric Appendicitis Score (PAS) [3], and the pediatric appendicitis risk calculator (pARC) [4], with each based on different sets of collected variables.

The Alvarado score is one of the most widely studied CPSs for the diagnosis of acute appendicitis in adult populations based on 8 variables with a total score of 10 [2], and it has since been studied in pediatric populations with varying results [33-44]. The PAS was developed specifically with slightly different variables for diagnosing appendicitis in children [3] and has been validated in multiple studies in various settings [42-54]. However, both the Alvarado score and PAS have been found to have limited clinical utility due to a large proportion of the scores falling into the intermediate risk group and failing to consider variability in clinical presentation based on factors such as age, sex, and duration of pain. This led to the development of the pARC score, which uses a multivariable prediction model that includes variables such as age, sex, and duration of pain to quantify the risk of appendicitis on a continuous scale [4], with promising results noted on various validation studies since its development [36,43,44,55-57].

However, few have compared multiple CPSs for pediatric appendicitis, and most existing studies are limited by the number of CPSs included [36,40,58], validated only in retrospective cohorts [59], did not include more recent CPSs such as the pARC score [40,59,60], and did not compare CPSs to clinician gestalt. Moreover, most of these studies were performed overseas with limited validation in children in Australia and New Zealand (ANZ) EDs [61]. This can result in inconsistent practice and an increasing number of children exposed to potentially unnecessary imaging and laboratory investigations.

### Objectives

The primary aim of the study is to externally validate various published CPSs for the risk of appendicitis in children aged 5 to <18 years presenting to ANZ EDs with acute abdominal pain and suspicion of appendicitis.

The secondary aims of the study are as follows:

1. To evaluate and compare the performance of various published CPSs:

(A) against clinician gestalt in ANZ for the likelihood of appendicitis at the time of ED assessment;

(B) in identifying, prospectively, the likelihood of complex appendicitis (defined as the presence of perforation, extraluminal fecalith, diffuse intraperitoneal pus, or a well-formed abscess) [62];

2. To describe the epidemiology and management of pediatric appendicitis, illustrating:

(A) The burden of disease in patients presenting with acute abdominal pain with the suspicion of appendicitis;

(B) The incidence and type of surgical procedures undertaken in the management of appendicitis in ANZ (laparoscopic appendectomy, open appendectomy, nonsurgical management with intravenous antibiotics, and delayed appendectomy);

(C) The imaging procedures undertaken in patients assessed for possible appendicitis, and their accuracy in the detection of histologically confirmed appendicitis or surgically confirmed appendicitis in cases where histopathological findings are unavailable.

This will improve the accuracy of diagnosis, reduce health care costs, rationalize the use of health care resources, and improve the management of childhood appendicitis. It will also provide a description of the current diagnostic and management approaches in ANZ for pediatric abdominal pain and appendicitis.

## Methods

### Overview

The study is an endorsed Paediatric Research in Emergency Departments International Collaborative (PREDICT) network [63] study with Perth Children’s Hospital (PCH) as the lead site. The study is a quantitative multicenter observational study that measures the performance accuracy of CPSs in identifying surgically or histologically confirmed appendicitis. Eligible CPSs (Multimedia Appendix 1) were identified in a rapid review by the study authors before study commencement [43]. Any CPS developed in either pediatric or adult populations was eligible except for: CPSs aiming only to differentiate simple from complex appendicitis or performed only for a subset of children (eg, preschool children and children with chronic illnesses); CPSs requiring variables unlikely to be routinely collected in a pediatric population identified a priori through consensus agreement by study authors (eg, rectal examination, biomarkers other than full blood picture, or C-reactive protein); and CPSs that included radiological parameters.

### Study Setting and Population

#### Overview

The study population will be all children presenting to participating ANZ EDs with abdominal pain. Study sites all have EDs that manage pediatric patients and have a pediatric on-site general surgical service with the capacity to provide input, admission, and surgical intervention if needed.

1. Perth Children’s Hospital (Western Australia, Australia)
2. Children's Hospital Westmead (New South Wales, Australia)
3. Gold Coast University Hospital (Queensland, Australia)
4. Monash Health (Victoria, Australia)
5. Queensland Children's Hospital (Queensland, Australia)
6. Royal Children’s Hospital (Victoria, Australia)
7. Royal Hobart Hospital (Tasmania, Australia)
8. Sunshine Coast University Hospital (Queensland, Australia)
9. Sunshine Hospital (Victoria, Australia)
10. Starship Hospital (Auckland, New Zealand)

#### Participant Inclusion Criteria

To be eligible to participate in the study, a participant must meet the following criteria:

1. Age 5 to <18 years,
2. Presentation with generalized or right-sided abdominal pain,
3. Duration of pain for ≤7 days (ie, ≤168 h),
4. Clinician’s concern for the possible diagnosis of appendicitis as defined by any one of the following:
5. Investigations performed (blood sample, imaging, or both, including external investigations before ED attendance),
6. Surgical consultation to assess the patient for possible appendicitis,
7. Senior ED clinician consultation to assess the patient for possible appendicitis,
8. Period of observation in ED to reassess patient for possible appendicitis.

For those hospitals with mixed ED and limited pediatric surgical capacity to operate on children under a certain age, the site-specific “pediatric” age range will be applied (eg, >10 years and <16 years). The age range of 5 to < 18 years was chosen due to the rarity and the atypical presentation of appendicitis in younger children [64], and many CPSs required variables on history or examination that might be difficult to determine accurately in younger children. Acute abdominal pain was defined as the duration of pain for ≤7 days in keeping with a previous study looking at abdominal pain and appendicitis [65], and a previous feasibility study found several patients with pain lasting for more than 5 days diagnosed with appendicitis [43].

#### Participant Exclusion Criteria

The following conditions exclude participation in the study:

1. Abdominal trauma that required medical review within the preceding 7 days,
2. Previous significant abdominal surgery (eg, appendectomy, short gut, ileostomy, and Hirschsprung disease),
3. Chronic illnesses that may affect the abdomen (including inflammatory bowel disease, chronic pancreatitis, cystic fibrosis, and sickle cell anemia),
4. Pregnancy,
5. Inability to obtain accurate history (eg, parent or guardian unavailable, language other than English and where no interpreter was available, or patients who are nonverbal due to pre-existing medical condition).

### Outcome Measures

#### Overview

The primary outcome is the presence of appendicitis; this will be confirmed through the histopathology report when available, or the operation report if not available. Patients diagnosed with appendicitis but managed nonoperatively will be followed up to confirm the presence of appendicitis in cases where interval appendectomy was performed or excluded from analysis in cases treated conservatively with antibiotics only. A nonappendicitis diagnosis will be confirmed on follow-up via either recording of the primary diagnosis on hospital discharge if admitted to hospital, or contact at 30-60 days to ensure no progression in symptoms or representation to hospital for those discharged directly from the ED. The area under the receiver operating characteristic (ROC) curve (AUC) will be calculated for the various published CPS for the primary outcome of appendicitis.

Secondary outcomes include rates of uncomplicated and complicated appendicitis (defined as the presence of perforation, extraluminal fecalith, diffuse intraperitoneal pus, or a well-formed abscess) [62], negative appendectomy cases (based on histopathological findings), and missed appendicitis cases. These will be confirmed through the histopathology report when available, and the operation report if not available. To identify missed appendicitis cases, families of children who did not undergo appendectomy and were discharged directly from the ED will be contacted within 30-60 days of hospital discharge to assess for postdischarge status and whether their child had an appendectomy in the interim. For families unable to be contacted, medical records will be reviewed at 60 days to assess for representation. Other secondary outcome measures include length of hospitalization, rates of investigations, and rates of interventions.

#### Patient Recruitment and Data Collection

All suitable patients with abdominal pain will be identified on ED presentation and approached by a research nurse (if available) or the treating clinician for consent for (1) study participation, data access, and collection and (2) follow-up contact. Patients who are not captured on the index visit (ie, “missed” recruitment) will be identified by the research team through a review of the daily ED attendance record using the terms “abdominal pain,” “appendix” or “appendicitis” on triage text or discharge diagnosis text “appendicitis” or “abdominal pain.”

Basic demographic data of missed patients (including any interventions performed and management) will be recorded on a separate datasheet and collected at the earliest possible time. Missed cases, if assessed by the research team to be eligible, will have a retrospective case report form completed by the treating clinician, if possible, and the family will be contacted via phone or text message to obtain informed verbal consent for inclusion retrospectively for (1) study participation and data access and collection and (2) follow-up contact. Follow-up will not be conducted for patients who are admitted to the hospital under an inpatient medical or surgical team or those who experience severe morbidity, such as a new diagnosis of chronic illness or permanent disability, or are deceased. Participants who decline consent for data access and collection will be excluded from the study.

The treating clinician will complete, at the time of the ED visit, or retrospectively if missed initially, a case report form recording:

- demographic data (sex and age),
- eligibility (inclusion and exclusion criteria),
- caregiver verbal consent,
- the clinician's seniority and their perceived likelihood of appendicitis on a Visual Analog Scale from 0 being “Extremely unlikely” to 10 being “Extremely likely” and whether this perceived likelihood was formed before or after investigations (bloods, imaging, or both),
- history of the presenting complaint (location of initial pain, duration of pain, highest pain score, highest reported temperature during this illness, right lower quadrant (RLQ) or right iliac fossa (RIF) pain on arrival to ED, pain migration to RLQ or RIF, progression of pain, gradual onset of pain, pain pattern, history of pain with walking, anorexia, nausea, vomiting, difficulty with micturition, pothole tenderness, family history of appendicitis, respiratory tract infection in last 2 weeks, and bowel habit), and
- examination findings (RLQ tenderness, RIF tenderness, tenderness worst in RLQ, tenderness outside RLQ, presence of hop, cough, or percussion tenderness, degree of rebound tenderness, abdominal guarding, Rovsing sign, nature of bowel sounds, palpable abdominal mass, and abdominal rigidity).

Clinician management will proceed independently of study participation.

All data collected at sites will be transcribed to a REDCap (Research Electronic Data Capture; Vanderbilt University) database [66]. The following additional data will be collected from the patient's medical records: blood test results (white cell count, absolute neutrophil count, neutrophilia, and C-reactive protein), urinalysis (presence of leukocytes or nitrites), which will then allow the various CPSs to be automatically calculated in REDCap.

Between 30-60 days after the ED visit, the site research team will collect the following parameters in all eligible patients through a medical record review:

- detailed demographics (postcode, Medicare status, race or nationality, and referral source),
- prehospital management,
- management and imaging were undertaken both pre-ED in the community and the study hospital,
- time-related data (times of triage, clinician evaluation, ED, and hospital discharge),
- duration of ED and hospital stay,
- admission status and specialty unit consultations, including intensive care admission,
- medication use in ED, including antibiotics and analgesia,
- related surgical and nonsurgical interventions,
- other significant pathology or adverse events, including mortality, and
- disposition.

#### Follow-Up

All eligible patients will be contacted for a follow-up survey except when (1) the parent or guardian declined follow-up on the initial presentation, (2) the participants were admitted under an inpatient medical or surgical team with no further representations to ED within the follow-up period of 30 days, or (3) participant identified by the site research team to have experienced clinically significant adverse events. Any approved parental contact will occur via a telephone call, email, or text, with scripts for each contact method provided to participating sites. Follow-up emails will be automatically generated and sent out twice during the follow-up period with identifiable data accessible only to the local study site and the central coordinating study team, and will be removed from the REDCap database at 61 days postenrollment date by the local study research team. A maximum of 3 contact attempts will be made. If more than 60 days have elapsed from the time of presentation, or if there have been three failed contact attempts, the patient follow-up will be regarded as unsuccessful. The medical record for patients unable to be contacted will be reviewed and if sufficient information is documented in the medical record, this information may be used to substitute for the failed contact.

The follow-up survey will determine the following:

- if the child had any problems with abdominal pain since being discharged from the ED,
- if the child missed any childcare or school because of abdominal pain,
- if the carers took any time off work because of the child’s ongoing pain, and
- if the child has been seen by another doctor, health professional, or hospital since the visit to the ED.

It will also collect the following details: details of ongoing problems; the number of missed days of school or childcare; the number of days off work; types and numbers of medical reviews; additional medical diagnoses; need for and duration of ED stay and hospital admissions including investigations (with a review of the relevant medical record); and related surgical interventions. Any eligible participants whose parent or guardian consented for data access and collection but declined follow-up will have their data collected and analyzed, but will not be contacted for follow-up. If participants withdraw consent and discontinue participation in the study at any time, there will be no effect on their medical care or access to treatment. If a participant withdraws before completing the study follow-up period, any known reason for withdrawal will be documented in the database. All information already collected as part of the study will be retained for analysis, but no further efforts will be made to follow up or obtain additional information regarding the participant. As an observational study, there are no anticipated adverse events related to the research. During the follow-up contacts, if any medical issues are identified by research staff, they will be referred to the managing clinicians (for patients in hospital), or the site investigator (for questions during follow-up), who will refer, if required, the patient to their general practitioner or ED for review.

To minimize information bias, the calculation of the CPSs will be performed by the research team and not by the treating clinician. If the study participant is transferred from a nonstudy site to a study site, the data collection form will be completed by the initial treating physician at the study site; if the study participant is transferred from a study site to a second study site, this will ideally be completed by the initial treating physician at the first study site.

### Sample Size and Power Calculation

Based on a feasibility study performed at PCH ED before the multicenter study [43], we estimate a true appendicitis rate of 30% in the multicenter study, and that all the CPSs will be calculable in at least half of the patient cohort. We aim to have a sample size of 2400 children with undifferentiated abdominal pain enrolled, which will result in all CPSs being calculable in a minimum of 360 enrolled cases of true appendicitis. This will provide a level of precision such that a 95% CI around the AUC will range from 0.016 for an AUC estimate of 0.95-0.032 for an AUC point estimate of 0.75 [67]. It will also provide more than 85% power for a 2-sided test, with α=.05, to find a difference of .05 between 2 AUCs if both values are ≥0.80, assuming the correlation of risk scores amongst appendicitis cases and that amongst noncases have an average of at least 0.5 [68]. Under this same assumption, if both AUC values are ≥0.9, the planned sample numbers would provide more than 90% power to detect a difference of 0.04.

### Statistics and Interim Analysis

We will follow Standards for the Reporting of Diagnostic accuracy studies (STARD) 2015 guidelines for statistics [69] and the Strengthening the Reporting of Observational Studies in Epidemiology (STROBE) guidelines [70], and validation of CPSs will be performed and reported in according to the 2014 updated methodologic standards for interpreting clinical decision rules in emergency medicine [71]. The AUC will be calculated for the various published CPSs and the clinician's gestalt for the outcome of appendicitis. The sensitivity, specificity, negative predictive value, positive predictive value, negative likelihood ratio, positive likelihood ratio, and missed appendicitis rate will be calculated based on the published cutoffs for each score [2-31]. For the pARC, subanalysis will be performed for the following pARC categories based on previous validation studies [4,56]: ≤5%, 6%-15%, 16%-25%, 26%-50%, 51%-75%, 76%-90%, and >90% for pediatric EDs and <5%, 5%-14%,15%-24%, 25%-49%, 50%-74%, 75%-84%, and ≥85% for mixed ED. For clinician’s gestalt, subanalysis will be performed for each point on the Visual Analog Scale. The AUC will also be calculated for complex appendicitis to assess the optimal cutoffs for each CPS to detect complex appendicitis.

The best-performing CPS will be identified by the highest AUC. Sensitivity and positive predictive value will be calculated to establish the CPS’s ability to identify high-risk patients, and the misclassification rate (proportion of patients identified as low-risk who had acute appendicitis) and negative likelihood ratio will be calculated to establish the CPS’s ability to identify low-risk patients. Accuracy will be reported as the proportion of patients not misclassified as either low-risk or high-risk as defined by published cutoffs. Baseline analyses of patient characteristics based on the risk groups as calculated by the best-performing CPS will be conducted by presenting simple counts and percentages with interquartile ranges.

### Ethical Considerations

The study received ethics approval from the Child and Adolescent Health Service Human Research Ethics Committee (RGS5295) for Australian sites and from the Northern A Health and Disability Ethics Committee for the New Zealand site (2024 FULL 19738). It was prospectively registered with the Australian New Zealand Clinical Trials Registry (ACTRN12622001293752) on October 6, 2022. Each participating site obtained site governance approval as per their ethics and governance committees. Informed consent will be obtained for all patients, with a waiver of consent for patients after 3 unsuccessful attempts to contact the parent or guardian for retrospective consent. Files containing private or confidential data will be stored only in secured locations accessible only by appropriate designated members of the research team, with all data de-identified before data entry onto the REDCap database. There is no compensation provided to participants.

## Results

**Figure 1:**
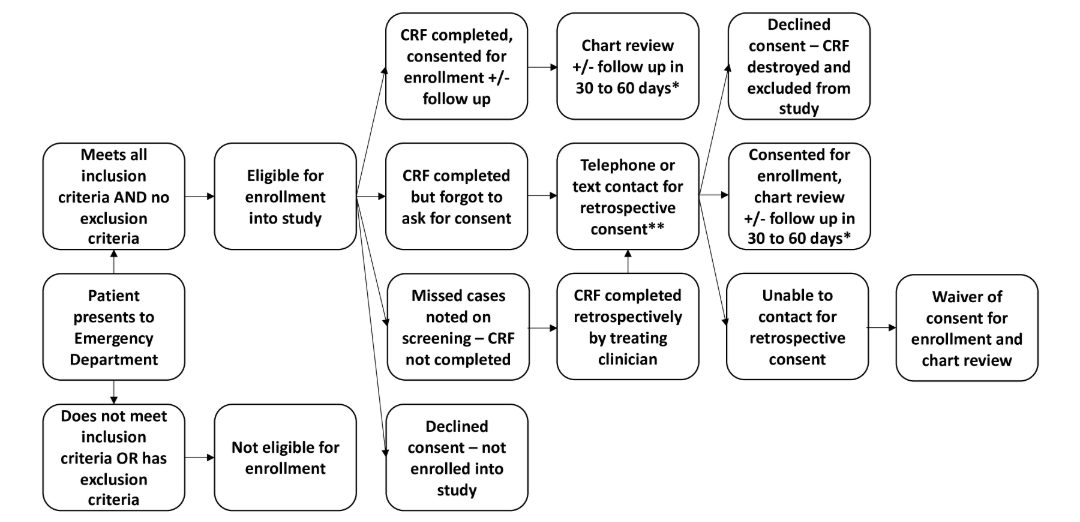
Study process and timeline. CRF: case report form. *Dependent on consent and need for follow up. **Contact for retrospective consent can be performed concurrently with follow up in 30-60 days.

The study recruitment process and timeline are shown in Figure 1.

This study received funding in July 2023 and started enrolment in August 2023. At the time of protocol manuscript submission in October 2024, we have enrolled and completed follow-up on 1227 participants with recruitment and data collection expected to be completed by mid-2025. Data analysis is expected to be completed by late 2025, and the expected results to be published in early 2026.

## Discussion

### Principal Findings

The pARC score was the best-performing CPS in the feasibility study through more accurate risk identification and stratification [43], and we hypothesize the pARC score will likewise have higher diagnostic accuracy in our multicenter study compared to other published CPSs.

### Comparison With Previous Work

The inclusion of 30 CPSs and the prospective nature of the study, with a planned study size of 2400 patients would make it one of the largest prospective validation studies in children, comparing the largest number of CPSs compared with previous studies [36,39,40,43,44,59,60]. A total of 10 EDs across ANZ will contribute data, including a mix of community and tertiary pediatric centers, which will allow the study's findings to be broadly generalizable across ANZ EDs compared to previous ANZ studies in single centers [6,43,44].

### Strengths and Limitations

Our study will allow the simultaneous comparative application and validation of 30 CPSs for diagnosing pediatric appendicitis outside their derivation settings. In addition to a high recruitment rate, the study will depend on high follow-up rates to ensure that our results accurately represent the whole population of children presenting to the ED with suspected appendicitis, as all patients lost to follow-up will be excluded from data analysis for CPS calculation. Ideally, all patients with abdominal pain would undergo investigations or appendectomy to determine the presence or absence of appendicitis; however, this would increase the rate of unnecessary investigations and potentially the rate of negative appendectomies should investigations be inconclusive. Therefore, a similar methodology to a previous PREDICT study looking at clinical decision rules for head injuries [72] was adapted using follow-up telephone or email contact to establish the presence or absence of appendicitis after discharge from the ED. It is also possible some patients with appendicitis would be treated conservatively with antibiotics and excluded from the study; however, this would represent a small number of patients as this is an uncommon practice in ANZ with only one patient in the feasibility study treated conservatively after a radiological diagnosis of appendicitis due to parental preference [43].

The predictor variables for the 30 CPSs were derived solely from original publications [2-31] and amalgamated into a single case report form developed by the study authors, which may introduce an element of interpretation in terms of the exact clinical terminology used in ANZ ED settings. The difficulty and variability of prospective clinician data collection in busy EDs in completing the case report form may result in a significant proportion of patients with multiple missing variables to calculate all CPSs; however, the feasibility study demonstrated the viability of the case report form used with all study CPSs being calculable in the majority of the patient cohort (ranging from 52% to 93.3%) based on constituent variables available [43]. Implementation of an appropriate method, such as multiple imputation, will be considered to minimize potential biases that may arise from missing data in the multicenter study [73].

Rates of computerized tomography imaging in ANZ EDs are lower than in the United States [74] due to concerns about radiation exposure and associated lifetime risk of malignancy and the more prevalent use of ultrasound in pediatric abdominal pain in Australian EDs [1]. The validation of CPSs in a different setting from those in which most of the CPSs are derived may be a potential key strength of the study and allow study findings to be generalizable across ANZ EDs and similar settings. Finally, one key strength of our study is its assessment of the accuracy of clinician gestalt in diagnosing pediatric appendicitis and comparison with to the best performing CPS, which may provide more compelling evidence to support and encourage the use of CPSs among clinicians for guiding clinical management of suspected appendicitis as part of an ED clinical pathway [75].

### Conclusion

The study findings and subsequent implementation of CPSs in ANZ EDs have the potential to reduce health care costs, rationalize the use of health care resources, and improve management and outcomes of childhood appendicitis. Implementation of the pARC score in the clinical management pathway for suspected appendicitis at PCH ED has recently been established following the results of the feasibility study, and a postimplementation study is planned to be performed in the future to assess for improvement in patient outcomes through impact analysis testing.

## Appendix

Multimedia Appendix 1 Clinical prediction scores and variables were collected.

Multimedia Appendix 2 Peer review report by the Emergency Medicine Foundation (EMF) Queensland Research Program (Australia).

## Abbreviations

ANZ: Australia and New Zealand
AUC: area under the receiver operating characteristic curve
CPS: clinical prediction scores
ED: emergency department
pARC: pediatric appendicitis risk calculator
PAS: Pediatric Appendicitis Score
PCH: Perth Children’s Hospital
PREDICT: Paediatric Research in Emergency Departments International Collaborative
REDCap: Research Electronic Data Capture database
RIF: right iliac fossa
RLQ: right lower quadrant
ROC: receiver operating characteristic
STARD: Standards for the Reporting of Diagnostic accuracy studies
STROBE: Strengthening the Reporting of Observational Studies in Epidemiology

## Data Availability

The datasets generated or analyzed during this study are available from the corresponding author on reasonable request.
