# Supplementary material for: Validation and Comparison of Pediatric Appendicitis Scores and Management Strategies (Project SPASMS): Protocol for a Prospective Multicenter Observational Study
Source: JMIR Res Protoc. 2025 Jun 27;14:e67941. doi: 10.2196/67941 (PMC12254709; doi:10.2196/67941)
Supplement: Multimedia Appendix 1 [file resprot_v14i1e67941_app1.docx]

**Multimedia Appendix 1**

Clinical prediction scores and variables were collected.

| Clinical prediction score | Patient factors | | | History of pain | | | | | | | | Signs or symptoms | | | | Examination findings | | | | | | | | | | Biochemical findings | | | |
| --- | --- | --- | --- | --- | --- | --- | --- | --- | --- | --- | --- | --- | --- | --- | --- | --- | --- | --- | --- | --- | --- | --- | --- | --- | --- | --- | --- | --- | --- |
| Variable | Age | Sex | Foreigner^a^ | Duration | Severity | Location | Migration | Worsening | Intermittent | Gradual onset | Pain with walking | Anorexia | Nausea or vomiting | Diarrhea | Dysuria or frequency | RLQ^b^ or RIF^c^ tenderness | Maximal tenderness in RLQ | Cough, percussion, or hop tenderness | Guarding | Rovsing sign | Abnormal Bowel sounds | Rigidity | Tenderness outside RLQ | Rebound tenderness | Fever | White cell count | Neutrophil count | C-Reactive protein | Urinalysis |
| Appendicitis Inflammatory Response |  |  |  |  |  |  |  |  |  |  |  |  | ✓ |  |  | ✓ |  |  |  |  |  |  |  | ✓ | ✓ | ✓ | ✓ | ✓ |  |
| Alvarado |  |  |  |  |  |  | ✓ |  |  |  |  | ✓ | ✓ |  |  | ✓ |  |  |  |  |  |  |  | ✓ | ✓ | ✓ | ✓ |  |  |
| APpendicitis PEdiatric Score (APPE) | ✓ | ✓ |  | ✓ |  |  |  |  |  |  |  |  | ✓ |  |  | ✓ |  |  |  |  |  |  |  | ✓ |  | ✓ | ✓ | ✓ |  |
| Anorexia, migratory Pain, local Peritonism, Elevated C-reactive protein, Neutrophilia and male gender (Dude) (APPEND) Score |  | ✓ |  |  |  |  | ✓ |  |  |  |  | ✓ |  |  |  |  |  |  |  |  |  | ✓ |  |  |  |  | ✓ | ✓ |  |
| Appendicitis Likelihood Model |  |  |  |  |  |  |  |  |  |  |  |  |  |  |  | ✓ |  |  | ✓ |  |  |  |  | ✓ |  | ✓ | ✓ |  |  |
| Children Appendicitis Score |  |  |  |  |  |  |  |  | ✓ |  |  |  |  |  |  | ✓ |  | ✓ | ✓ |  |  |  |  |  |  | ✓ |  | ✓ |  |
| Christian |  |  |  |  |  |  |  |  |  |  |  |  | ✓ |  |  | ✓ |  |  |  |  |  |  |  |  | ✓ | ✓ | ✓ |  |  |
| Eskelinen |  |  |  | ✓ |  | ✓ |  |  |  |  |  |  |  |  |  | ✓ |  |  |  |  |  | ✓ |  | ✓ |  | ✓ |  |  |  |
| Fenyo-Lindberg |  | ✓ |  | ✓ |  |  | ✓ | ✓ |  |  |  |  | ✓ |  |  |  |  | ✓ |  |  |  | ✓ | ✓ | ✓ |  | ✓ |  |  |  |
| Izbicki |  | ✓ |  | ✓ |  |  | ✓ |  | ✓ |  |  |  |  |  |  |  |  |  | ✓ |  |  |  |  | ✓ |  | ✓ |  |  |  |
| Jawaid |  | ✓ |  |  |  | ✓ | ✓ |  |  |  |  | ✓ | ✓ |  |  |  |  |  | ✓ |  |  |  |  | ✓ | ✓ | ✓ | ✓ |  |  |
| Lintula |  | ✓ |  |  | ✓ | ✓ | ✓ |  |  |  |  |  | ✓ |  |  |  |  |  | ✓ |  | ✓ |  |  | ✓ | ✓ |  |  |  |  |
| Low Risk Appendicitis Rule |  |  |  |  |  |  |  |  |  |  |  |  | ✓ |  |  |  | ✓ |  |  |  |  |  |  |  |  |  | ✓ |  |  |
| Low Risk Appendicitis Rule Refinement |  |  |  |  |  |  |  |  |  |  | ✓ |  |  |  |  |  | ✓ | ✓ |  |  |  |  |  |  |  |  | ✓ |  |  |
| Modified Alvarado Score by Shera^d^ |  |  |  |  |  |  | ✓ |  |  |  |  | ✓ | ✓ |  |  | ✓ |  | ✓ |  |  |  |  |  |  | ✓ | ✓ | ✓ |  |  |
| Alvarado Score without bloods |  |  |  |  |  |  | ✓ |  |  |  |  | ✓ | ✓ |  |  | ✓ |  |  |  |  |  |  |  | ✓ | ✓ |  |  |  |  |
| Modified Alvarado Scoring System |  |  |  |  |  |  | ✓ |  |  |  |  | ✓ | ✓ |  |  | ✓ |  |  |  |  |  |  |  | ✓ | ✓ | ✓ |  |  |  |
| Modified Lindberg |  | ✓ |  | ✓ |  |  | ✓ | ✓ |  |  |  |  | ✓ |  |  |  |  |  |  |  |  | ✓ | ✓ | ✓ | ✓ | ✓ |  |  |  |
| Paediatric Appendicitis Score without bloods |  |  |  |  |  |  | ✓ |  |  |  |  | ✓ | ✓ |  |  | ✓ |  | ✓ |  |  |  |  |  |  | ✓ |  |  |  |  |
| Ohmann^e^ |  |  |  |  |  |  | ✓ | ✓ |  |  |  |  |  |  | ✓ | ✓ |  |  |  |  |  | ✓ |  | ✓ |  | ✓ |  |  |  |
| Paediatric Acute Appendicitis Score |  |  |  |  |  | ✓ |  |  |  |  |  |  | ✓ |  |  |  |  |  | ✓ |  |  | ✓ |  |  |  |  |  |  |  |
| Paediatric Appendicitis Score |  |  |  |  |  |  | ✓ |  |  |  |  | ✓ | ✓ |  |  | ✓ |  | ✓ |  |  |  |  |  |  | ✓ | ✓ | ✓ |  |  |
| Pediatric Appendicitis Risk Calculator | ✓ | ✓ |  | ✓ |  |  | ✓ |  |  |  | ✓ |  |  |  |  |  | ✓ |  | ✓ |  |  |  |  |  |  |  | ✓ |  |  |
| Predictive Factor |  |  |  |  |  |  |  |  |  | ✓ |  |  |  |  |  | ✓ |  | ✓ | ✓ | ✓ |  | ✓ |  | ✓ |  |  | ✓ |  |  |
| Ramathibodi Appendicitis Score (RAMA-AS) |  |  |  |  |  |  | ✓ | ✓ |  |  | ✓ |  |  |  |  |  |  | ✓ |  |  |  |  |  | ✓ | ✓ | ✓ | ✓ |  |  |
| Ramirez |  | ✓ |  |  |  | ✓ |  |  |  |  |  |  |  | ✓ |  |  |  |  | ✓ |  |  |  |  | ✓ |  | ✓ | ✓ |  |  |
| Reynolds |  |  |  |  |  | ✓ |  |  |  |  |  |  | ✓ |  |  | ✓ |  |  | ✓ |  |  |  | ✓ |  |  |  |  |  |  |
| Raja Isteri Pengiran Anak Saleha Appendicitis (RIPASA) Score |  | ✓ | ✓ | ✓ |  | ✓ | ✓ |  |  |  |  | ✓ | ✓ |  |  | ✓ |  |  | ✓ | ✓ |  |  |  | ✓ | ✓ | ✓ |  |  | ✓ |
| Simplified Appendicitis Score |  |  |  |  |  |  | ✓ |  |  |  |  |  |  |  |  | ✓ |  |  |  |  |  |  |  | ✓ | ✓ | ✓ |  |  |  |
| Ting |  |  |  |  |  |  | ✓ |  |  |  |  | ✓ | ✓ |  |  | ✓ |  |  |  |  |  |  |  | ✓ | ✓ | ✓ | ✓ |  |  |
| van den Broek |  | ✓ |  | ✓ |  |  |  |  |  |  |  |  |  |  |  |  |  |  |  |  |  |  |  | ✓ | ✓ | ✓ |  |  |  |

^a^Medicare status used as proxy for foreigner status.

^b^RLQ: Right Lower Quadrant.

^c^RIF: Right Iliac Fossa.

^d^Modification of previous scores – validation study only.

^e^Validation study of previous score.
