# Supplementary material for: Validation and Comparison of Pediatric Appendicitis Scores and Management Strategies (Project SPASMS): Protocol for a Prospective Multicenter Observational Study
Source: JMIR Res Protoc. 2025 Jun 27;14:e67941. doi: 10.2196/67941 (PMC12254709; doi:10.2196/67941)
Supplement: Multimedia Appendix 2 [file resprot_v14i1e67941_app2.pdf]

Project Grant Round 39
Response to Review - EMPJ-272R39-PHILLIPS
Application No. EMPJ-272R39-2023 From Dr Natalie Phillips
Form Submitted 2 Jun 2023, 11:03PM AEST

Applicant Response to Review

Dear Applicant

The reviewers have assessed and commented on your application's scientific and technical merit with regard to the review criteria below. You now have the opportunity to respond to the reviewers' comments.

Please provide your response to review by **midnight Friday 2 June 2023 (AEST)**.

Please note that the online assessment form does not auto-save and times out after 30 minutes. Please save the form regularly or prepare your response in a Word document and paste into the online form at the end.

The purpose of a grant applicant's response to review is to address the questions and concerns raised by reviewers and to convince them of the feasibility and fundability of the application. Reviewers are your peers who are making themselves available to assess the application and your response to review. This response will be used by the EMF Research Evaluation Panel when scoring your application.

While responding to review, please keep the following points in mind:

- Address concerns raised by reviewers systematically with a well-thought out plan that is specific, measurable, attainable, and relevant.
- Keep your response concise, jargon-free, objective and factual, and provide evidence to support the response.
- Avoid being overly defensive in tone or dismissing the reviewer as being incorrect. A clarification of differences of opinion, overcoming misunderstanding or addressing weaknesses in the proposal are all part of a constructive peer review process.

EMF expects that applicants comply with the Australian Code for the Responsible Conduct of Research.

Review Criterion 1 - Scientific Quality (30%)

Definition

The reviewer was asked to assess how well your grant application meets these requirements:

- The proposed research is robust and consistent with and appropriate to the aims of the project, i.e. regarding design, methods, outcome measures, data sources, variables, sample size, analysis etc.
- The overall strategy is well-reasoned and appropriate to accomplish the research aims of the project.
- There is evidence of stakeholder engagement to help support the implementation of the research design and methods employed.
- If the proposed research involves recruitment of participants (e.g. patient, staff) or accessing data, has the applicant provided sufficient evidence for feasibility?

Reviewer 1 Assessment

Strengths:

Well put-together study.

Need for ANZ prospective validation is justified by the literature and established regional practice variations from the derivation and initial validation sites.

Weaknesses:

Although minor, the background information lacks some detail as to the extent of existing prospective validation studies of the scores to be examined in this study.

Sample size calculations are specified based on PCH attendances, but it is unclear how this translates to the Qld contribution. How many patients are you anticipating from the QLD sites and what proportion of the study population is this likely to be?

Comments/Suggestions:

Please detail the extent of existing prospective validation studies in other regions.

Applicant - Response to Reviewer 1

Many thanks for your review and the questions/considerations raised.

Existing validation studies:

To our knowledge, there are no robust multicentre, prospective validation studies in Australia considering multiple CPSs, including the 3 most well- known scores, pARC, Alvarado and PAS, although such scores are variably used in clinical practice. Prospective validation and retrospective studies have been conducted in Europe and North America with varied results ((Van Amstel et al J Gastrointestinal Surgery, 2019; RIFT, Lancet Child and Adolesc 2020; Systematic reviews: Ebell et al, 2014: ; Kulik, J Clin Epidemiol 2013) and variably incorporated into international hospital management pathways. Some international EDs are even combining scores in their management protocols (e.g. Children's Hospital of Philadelphia). This highlights the importance of robustly determining how the CPSs perform in our context prior to integration into routine guidance.

Alvarado and PAS have also been considered outside of Australia, in both retrospective and prospective reviews, with varied results with PAS performing better in some populations, Alvarado in others, and neither well enough in other studied populations. Concerns have also been expressed about the derivation populations of each score (Alvarado not paediatric specific and derived from retrospective data, neither derived from undifferentiated ED populations, both included limited centres). pARC while prospectively developed and validated in a multicentre context, has had limited validation to date outside of the USA. In internal validation, the pARC had a greater ROC curve AUC compared to the PAS (0.85 [95%CI: 0.83-0.87] vs 0.77 [95%CI: 0.75-0.80], respectively) and quantified more patients in either high or low risk allowing for more certainty in diagnosis for ED and surgical clinicians. In an external validation, conducted at 11 centres in the USA, the pARC demonstrated a ROC curve AUC of 0.89 (95% CI 0.87-0.92), superior to the PAS 0.80 (95%CI 0.77-0.82).

The RIFT study in the UK (RIFT study Group, Lancet Child Adolesc Health, 2020) compared multiple routinely collected scores including PAS and Alvarado, but didn't include the pARC score (published 2018). The best performing CPS was the Shera CPS (little known in Australia). Furthermore, the RIFT study relied on data that could be extracted from the routine clinical record (and thus could not validate several scores); SPASMS will collect study variables prospectively on a specific study proforma to ensure that all relevant variables are collected. While SPASMs is focussed on the 3 most commonly well- known scores, variables will be collected to allow additional lesser well known CPSs identified to be assessed (such as the Shera).

Sample size:

The Queensland sites included in this grant are expected to contribute at least 30% to the total numbers of enrolled participants in the SPASMS study based on previous research experience, enrolment rates, and the combined census of the non- Queensland hospitals

committed to this project. Queensland Children's, Gold Coast University and Sunshine University Hospitals have a combined annual paediatric (<18) census of ~140,000 patients per annum (nearly 80,000 at QCH). Perth Children's Hospital (site of the pilot study, hence the sample size estimations from this study) sees ~ 70,000 children per annum. While positive rates of appendicitis may vary (i.e. number of true cases among those included in the study) , there is no reason to suspect significant variation in the numbers of true cases of appendicitis per 10, 000 presentations across Australia. Based on recruitment at PCH (with a higher number of positive appendicitis cases than previously expected), target numbers should be easily achieved by all involved sites.

Reviewer 2 Assessment

Strengths:

The PREDICT network has a strong track record in generating evidence to fill a local gap, and, translating that evidence.

Weaknesses:

Plain language summary would be strengthened by including a stat about how common abdo pain is for paed presentations.

The applicant states that the "project is expected to improve diagnostic accuracy, reduce healthcare costs, rationalise the use of healthcare resources, potentially reduce unnecessary inter-hospital transfers and improve overall management of childhood appendicitis." How will this be achieved by simply externally validating a tool? There is no mention of how the study will translate to practice change.

Comments/Suggestions: Nil

Applicant - Response to Reviewer 2

Thank you for the feedback. We will aim to incorporate a statistic on how common abdominal pain is as a reason for ED presentation in future plain language summaries. Previous Australian work has shown 5-7% of children in the target age group presenting to ED have a primary complaint of abdominal pain (please see Criterion 2 feedback; response to reviewer 3).

Validating CPSs variably used across Australian centres will allow us to identify the best -performing score available for use in our setting. This, in combination with a better understanding of the variability and nature of the care of children presenting with possible appendicitis, and the role of clinician gestalt should assist with streamlining and standardising care, and provide national guidance (such as occurs in head injury). Reliable tools in our setting offer the opportunity to better identify the risk of appendicitis in paediatric patients with benefits to patients, clinicians and the health care system. Better and evidence-based identification of risk, should allow treating clinicians to tailor their care with more confidence. This care includes the need for further investigations, surgical referrals and at times, interhospital transfers. Assessing tool performance in our setting, also allows us to identify tools that don't perform well – these CPSs are used variably across Australia and New Zealand and it is important to ensure there is good evidence for use, prior to any implementation.

The research team, supported by the PREDICT research network, is fully cognisant that further steps beyond knowledge generation are required to achieve practice change, has a proven commitment to undertaking these steps and is well placed to do so. Most of the study team is actively engaged in the acute care environment, including as local, state and national clinical leaders and educators, institution and state guideline authors, and senior on floor clinicians.

Reviewer 3 Assessment

Strengths:

This is a multisite prospective observational study designed to improve the diagnosis of children aged 5-18yrs with appendicitis. By investigating the diagnostic accuracy of several Clinical Predictions Scores for Appendicitis, the researchers propose that the study will improve decision making and outcomes in children with acute abdominal pain with clinical suspicion of appendicitis, in comparison to other published CPSs and clinician gestalt in the ANZ ED setting. This will in turn reduce healthcare costs, rationalise the use of healthcare resources, potentially reduce unnecessary inter-hospital transfers and improve overall management of childhood appendicitis. The study is already being conducted (and is funded) outside of Queensland, and the proposed funding is to allow the study to occur in Queensland. The study will also allow the researchers to describe the current diagnostic and management approaches in Australian and New Zealand for paediatric abdominal pain and appendicitis.

Weaknesses:

This is a well-conceived, well-designed programme of research. There are some issues that require clarification.

What is the rationale for the 3 included sites? Eg – state "sites have been selected where the ED manages paediatric patients and has a paediatric on-site general surgical service with capacity to provide input and/or admission". There are other sites in QLD that have these characteristics, so what is the rationale for these sites specifically? I am not saying that the choice of sites is inappropriate, I am just wondering what the rationale is? Also, I note that most of the sites are in large, metropolitan locations, with no regional sites. Is there a possibility for inclusion of other sites (e.g., TUH)? There are some issues with sample size and selection.

The sample size calculations seem to relate to PCH (which I'm presuming is Perth Children's Hospital)? So I'm wondering what is the added benefit of including the Queensland studies? Can the researchers please relate this to QLD? What portion of the sample size is anticipated to be from Queensland, and then from each site in Queensland? As this is an observational study the sample size is not necessarily a limiting factor – you will recruit what is recruitable and analyses will be performed on that sample. But it would be good to demonstrate the anticipated sample size to be recruited from the participating QLD sites, for which this funding is being sought, the feasibility of this, and whether it will be possible to conduct site specific (or QLD-specific) on the QLD sample.

Is it anticipated to conduct stratified analyses to assist with the external validation ? That is, different cultures operate within different hospital sites and states, so it would be useful to demonstrate the validity of the CPS at the different sites (or at least withing different health systems. The stated sample size was calculated with respect to Perth Children's Hospital. One of the reasons to include QLD sites is to validate the CPS at other sites (different health systems, different hospitals etc). It is logical then to conduct site-specific (or at least state specific) analyses. This is not mentioned anywhere, and would improve the application (the sample size calculations would need to be adjusted accordingly, to demonstrate the feasibility of conducting the planned analyses at the state level, or even site level, if possible).

In the first paragraph of the sample size calculation there is mention that 8% of children presenting to ED with chief complaint of abdo pain have appendicitis. But in the 2nd para, it states that of the 4500 children anticipated to be screened, approx 1200 will be assessed for appendicitis, and 30% of these will have appendicitis (true +ve). Why are these different (8% vs 30%)?

The analyses are generally described (but not specifically). There are no specific analytical strategies mentioned (only that ROC will be calculated, with SN, SP, PPV and NPV – not how

these will be calculated). This is not particularly concerning but it would help demonstrate that the required epidemiological and statistical expertise is present within the team (or accessible to the team).

There is no mention of stratified analyses (i.e., validity of CPS in different age group / gender /ethnicity/ combination of these. This study would be greatly enhanced by adding this kind of sub-group analyses, as the researchers could then demonstrate CPS validity in different groups. Finally, the analysis section indicates that subgroup analyses will be conducted (specifically, the researchers state "for pARC, on the groups ≤5%, 6-15%,16-25%, 26-50%, 51-75%, 76-90%, and >90% for paediatric EDs and <5%, 5-14%,15-24%, 25-49%, 50-74%, 75-84%, and ≥85% for mixed EDs"). However, the sample size calculations do not refer to these subgroup analyses. Could the researchers please provide some reassurance that the anticipated sample will allow these planned analyses?

Feasibility – The researchers have allowed 3 months for "governance local site SSAs / approvals". I suggest this needs to be longer. This is assuming ethics has already been obtained. If ethics has not yet been obtained, then I would allow even longer.

Comments/Suggestions: Nil

Applicant - Response to Reviewer 3

Many thanks for the detailed critique and the opportunity to clarify the questions raised. We have endeavoured to answer these in the order raised.

Site selection:

Queensland sites were selected based on sites which 1. Had access a general paediatric surgical service with the capacity to admit +/- perform appendectomies (i.e. complete definitive patient care) 2. Had actively identified themselves as highly interested following EOIs sent by the lead site across the PREDICT network, pending funding availability – GCUH and SCUH expressed such an interest, QCH has been involved with the central study team since the early stages of SPASMs planning (Drs Phillips (ED) and Dr Patel (General Surgeon)). Sites were aware they would have to source their own funding for site participation in this study. The study team would welcome additional suitable Queensland sites (& could approach them), should supplemental funding be available and there be sufficient site interest.

Sample size calculations, rationale for including Queensland sites and inter-site variation:

The Queensland sites included in this grant are expected to contribute at least 30% to the total numbers of enrolled participants in this study based on previous research experience and the combined census of the non- Queensland hospitals committed to this project. Queensland Children's, Gold Coast University and Sunshine University Hospitals have a combined annual paediatric (<18 years) census of ~140,000 patients per annum (nearly 80,000 at QCH). Perth Children's Hospital (site of the pilot study, hence the sample size estimations from this study) sees ~ 70,000 children per annum. While positive rates of appendicitis may vary (ie number of true cases among those included in the study) , there is no reason to suspect significant variation in the numbers of true cases of appendicitis per 10, 000 presentations across Australia. Based on recruitment at PCH (with a higher number of positive appendicitis cases than previously expected), target numbers should also be easily achieved by all involved sites.

The inclusion of multiple locations/states (i.e.multi-site) will enhance the applicability of any result achieved in this validation study, and strengthen the findings. It is envisaged that a national guideline will be informed by this study (such as has occurred with head injury and bronchiolitis), and the inclusion of Queensland sites in this study will be crucial to ensuring findings are of direct relevance to Queensland children and clinicians. Queensland faces many similar issues to WA in terms of distance and the number of hospitals which see paediatric patients across the state; standardization streamlining of care with more

confident identification of, and better evidence behind which patients can be safely discharged home, which need direct referral to surgical services and which need further investigation and/or observation, may improve clinician confidence, better tailor resource use and potentially decrease the number of, and expedite necessary, interhospital transfers.

This study has additional benefits beyond the validation of the CPSs. It will allow us to better understand the population of children presenting with possible appendicitis and their management across Australia. This may offer further opportunities to streamline care, and hospital management pathways. A number of subanalyses including, geographical and site variation in epidemiology practice will be considered as part of this study (as has been done for other PREDICT multicentre studies eg head injury – Phillips et al, 2019, EMA); unfortunately there was not sufficient space to detail these in the included submission.

Stratified analyses, clarification of sample sizes, and clarification of analyses:

We apologise for any confusion in the application. A previous prospective study of abdominal pain on behalf of PREDICT (4 sites, Australian data, mixture of suburban and tertiary children’s hospital in Victoria and WA) found that 6.2% of ED presentations had the chief complaint of abdominal pain, and 8% of these children had a diagnosis of appendicitis (Lee, 2019) . The prospective pilot for the SPASMS study (WA, tertiary Children’s Hospital only) has shown a 30% incidence of appendicitis in a more targeted population where the clinicians had to have a suspicion of possible appendicitis. The inclusion criteria in the latter study were more select. This rate may differ between sites, however there is no reason to suspect that actual case numbers of appendicitis vary between Australian sites per 10,000 presentations. Based on recruitment at PCH (with a higher number of positive appendicitis cases than previously expected), target numbers should be easily achieved by all involved sites.

Most CPSs do not have stratified analyses based on group/gender/ethnicity/specific age group or location as part of their validation; and sample sizes (and number of positive cases of appendicitis) for the SPASMs study are similar to that for the original pARC validation (with groupings described). Data from the pilot study however, are undergoing further analyses from the supporting statistical team to further ensure sample size calculations are sufficient (and allow a buffer if an unexpectedly low incidence of true appendicitis occurs).

The suggestion of the value of stratified analyses though is very valid e.g. for age group/gender/ethnicity and in particular region/state analysis; we thank the reviewer for this and will discuss it with the statisticians.

Feasibility:

Ethical approval for study in already in place, and additional sites are being added to the original ethical approval under the National Mutual Agreement (NMA). All 3 participant sites are experienced in submitting governance approvals and would expect to be able to complete the required approvals within the three-month period.

Review Criterion 2 - Importance to Emergency Care (30%)

Definition

The reviewer was asked to assess how well your grant application meets these requirements:

Application leads to:

- Improvements in the field of emergency medicine to

    - enhance the patient experience and outcome, save lives, and/or
    - support staff providing services in Emergency Medicine and/or
    - support system sustainability
- Improved access to treatment, time to recovery, degree of full recovery
- Capacity to improve upon current best practice through knowledge/novelty, degree of innovation in methods, equipment, techniques, cross-disciplinary approaches, generation of new research output and impact for the patient and community

Application develops emergency medicine research capacity in Queensland, for example through:

- Inclusion of regional, rural or remote sites
- Inclusion of novice and early-career researchers in the team

Applications that are not entirely within the core area of emergency medicine, e.g. projects in population health or intensive care, must provide a strong justification of how they meet this selection criterion.

Reviewer 1 Assessment

Strengths:

This is an important and common area of emergency medicine. As outlined in the proposal, there is potential scope for this work to inform more efficient use of investigations and observation strategies.

Weaknesses:

Some of this work has been done before in other countries. However the investigators provide some justification as to why result might be different in ANZ.

Comments/Suggestions: Nil

Applicant - Response to Reviewer 1
As noted by the reviewer while some of this work has been done in other countries, it is important to understand how these rules perform in an Australian environment. Substantial variation in practice between Australia and other regions such as North America, has been noted in previous research (eg imaging in children's head and cervical spine injury where North America substantially higher imaging rates (Leonard 2019, Phillips 2021, Babl 2017). Limited validation of pARC outside US settings has occurred, and concerns have been expressed with the derivation populations of PAS and AS, although both are often used in paediatric settings.

To our knowledge this would be the first robust multicentre validation study on this topic in an Australian context

Reviewer 2 Assessment

Strengths:

Addressing an area of need to standardise ANZ clinical practice and enable evidence based practice and access to evidence based care for children with abdo pain.

Weaknesses:

Nil inclusion of rural / smaller regional sites, and one of the arguments made for the need for this study is that there are limited paediatric on-site general surgical services - all the study sites have these. So does the validation really address the argument made?

Nil mention of addressing facilitators and barriers to use of particular tools, if practice change following validation is the goal, these data should also be collected, from medical, surgical and ED nursing staff (who are often the advocates for/against particular treatment).

Comments/Suggestions:

Collect facilitators and barriers to use of a CDS for this patient group.

Applicant - Response to Reviewer 2
As noted by the reviewer, this study design includes only sites with access to general paediatric surgical services with the capacity to admit and/or perform appendectomies. This is to allow validation of the clinical decision scores as the primary outcome of the study is the presence of appendicitis which will be confirmed through the histopathology report when available, or operation report if not available. It is anticipated that sites needing to transfer patients will be involved in further research endeavours related to and ensuing from this particular project as the research team, are very mindful of the fact that many children are seen outside tertiary and large centres for emergency care of their abdominal pain and possible appendicitis.

The study team agrees that identification of barriers and facilitators is crucial to successful knowledge translation and practice change. PREDICT has undertaken such work previously such as in bronchiolitis and head injury. While beyond the scope of this particular grant application, it forms a key part of a parallel project being undertaken by the investigator team and Dr Wei Hao Lee as part of his PhD on the care of children with appendicitis and abdominal pain. This qualitative work is intended to involve Queensland participants. Understanding variation in practice within and between institutions is also crucial to any knowledge implementation efforts – this will considered within this grant application; and future work involving a broader range of hospitals.

Reviewer 3 Assessment

Strengths:

This study has high potential to improve emergency care. As above, the researchers propose that the study will improve decision making and outcomes in children with acute abdominal pain with clinical suspicion of appendicitis, in comparison to other published CPSs and clinician gestalt in the ANZ ED setting. This will in turn reduce healthcare costs, rationalise the use of healthcare resources, potentially reduce unnecessary inter-hospital transfers and improve overall management of childhood appendicitis.

There is also demonstrated effort in this application to develop emergency medicine research capacity. The applicants include a mix of highly experienced ED researchers, mid-career researchers and early career researchers from emergency and surgical fields. The applicants have specifically chosen junior Queensland researchers from different multi-disciplinary teams (CI Shellshear; AIs Moore, Davidson, Mulready)., citing their valuable clinical expertise, and have stated an explicit aim of the research is to provide support and encouragement to further their research involvement and experience.

Weaknesses:

This application could be improved by more explicit demonstration of the potential impact of this research. This is alluded to but could easily be strengthened by adding context, which would improve the application and increase the score for this criterion. That is, how many presentations per annum (or per month, or for a specified time period) in QLD (at each site, but at a minimum at QCH) for abdo pain, and then for Appendicitis? This would help to convey the magnitude of the issue and consequent health service utilisation / cost. This is required to demonstrate bang for buck.

As mentioned in a previous comment, the application could improve on this criterion by inclusion of a regional / remote site, but it's not a deal breaker.

Comments/Suggestions: Nil

Applicant - Response to Reviewer 3
It is difficult to accurately retrospectively obtain appendicitis rates from ED records, due to inaccuracies in the coding of abdominal pain and appendicitis, and the provisional nature of ED diagnoses. With that caveat, QCH data from 2022 showed that 2181 children aged 5-17 years inclusive had a primary diagnosis of abdominal pain and 286 (11%) a provisional diagnosis of appendicitis on leaving the ED.

Other Australian research has shown that abdominal pain is a common reason for presentation to Australasian EDs and accounts for approximately 5% of ED presentations in children aged 5-14 years (Acworth et al, 2009). A small prospective study of abdominal pain on behalf of PREDICT (4 sites, Australian data, mixture of suburban and tertiary children's hospital in Victoria and WA) found that 6.2% of ED presentations had the chief complaint of abdominal pain, and 8% (Lee, 2019) of these children had a diagnosis of appendicitis. This is consistent with previous international and local studies reporting an incidence of appendicitis of 1-8% of abdominal pain presentations (see application B1). The prospective pilot for the SPASMS study (WA, tertiary Children's Hospital only) has shown a 30% incidence of appendicitis in a more targeted population where clinicians suspect appendicitis.

Furthermore in the 4 site prospective study, eighty-two patients (14.8%, 95% CI 11.8-17.7%) had clinically significant abdominal pain (pre-defined as patients requiring; surgical procedure for appendicitis; other invasive treatment of other abdominal pathology; radiologically guided abscess drainage; conservatively managed complex appendicitis and/or admission for more than 48 hours for investigation of abdominal pain). One-month follow-up of included patients identified a significant burden on families through time off school/work for children and carers, and need for ongoing analgesia, irrespective of the final diagnosis, thus highlighting the community burden of this condition in Australia.

Review Criterion 3 - Investigator & Team (20%)

Definition

The reviewer was asked to assess how well your grant application meets these requirements:

- Investigator and team quality: Principal Investigators, Co-Investigators, and other collaborators are well-suited to the proposed research.
- Team track record in translating outcomes into practice and management.
- Capability relevant to the application and relative to opportunity:
    - If early-career investigators, does the application provide strategies to gain this experience and training?
    - If established investigators, does the application demonstrate how previous research has advanced their field/s?
    - Will the research environment in which the work will be done contribute to the probability of success?

(Please note, a CV for each investigator named in addition to the original submission must be provided - the upload facility is available at the end of the document.)

Reviewer 1 Assessment

Strengths:

Strong, networked team with good track record in delivering these types of studies.

Weaknesses:

It would be good to see some research capability development expressed with the Qld team membership.

Comments/Suggestions: Nil

Applicant - Response to Reviewer 1

Consideration has been given to research capability development, and will be approached on a number of levels.

Queensland investigator Team:

Several less experienced researchers have been included in the Queensland investigator team including a nurse practitioner who works between QCH and a suburban mixed ED; a clinical nurse educator (CNC) who works between a larger regional hospital (SCUH) and associated more rural hospitals (Nambour/Gympie); and two less research experienced ED senior medical officers (no grant application experience) at different sites.

Research Assistant Staff (ED based):

This study would employ local ED based research staff. These staff are often ED nurses with learned experience of the day to day problems facing ED clinicians. Participation in this project is expected to enhance these skills amongst the team and provide opportunities for national networking and education in the Paediatric ED research space. Regular education (including in person research development days) and monthly catch up/RA trouble shooting meeting occurs within the PREDICT network. QCH hosted a 2-day event last year with RAs from 13 sites across Australia; RCH Melbourne will host a similar day in July 2023.

Cross specialty team engagement: This research is expected to strengthen general paediatric surgical and ED collaboration, and establish a solid basis for future cross-speciality collaborative research.

Wider ED staff active participation in research:

Members of the general ED nursing team and within the CN and nurse educator group are often appointed as research champions for specific projects, according to interest and role. The intent would be to continue this for SPASMS. We have done this highly successfully with previous and ongoing projects, enhancing general team enthusiasm, and engagement in research and on occasion, taking on increasing research roles (Projects including acute behavioural disturbance, Sepsis, Burns RCTs; Observational studies such as cervical spine injury and sepsis projects).

Opportunities also exist within the project for trainees including ED fellows to be actively involved in this study. ED trainees often change terms every 6-12 months, and opportunities to be actively involved in such a project are often offered to trainees closer to project commencement. QCH for example has ED fellow champions for each Research Project we are involved in, which offers enhanced insight into research projects, and networking opportunities, and has led many trainees to become more broadly involved in research, including development and execution of their own projects.

Staff involved in research at our centres (nursing and medical) have previously taken research skills and enthusiasm to other facilities across Queensland (including non-tertiary centres) participating in other research projects including multicentre PREDICT studies. We would expect this to continue.

Networking opportunities:

As discussed above team members have the opportunity to be involved in monthly and bimonthly RA and investigator sessions, and be involved in the wider PREDICT (Paediatric Research In Emergency Departments International collaborative) network.

Reviewer 2 Assessment

Strengths:

A strong track record within a research network and high probability of successful completion.

Weaknesses:

Nil nursing staff on investigator team which may hinder true translation of any CDS.

Comments/Suggestions: Nil

Applicant - Response to Reviewer 2

The value of the multidisciplinary team, including the ED nursing team is well recognised in Paediatric Emergency Research, the Qld investigator team and PREDICT (several members of the PREDICT Executive have a nursing background, and a number have achieved their PhD through PREDICT affiliated research). One of these nurses is part of the wider national SPASMS team, Dr Sharon O'Brien, and will be well able to assist in mentoring more research junior members of the research team, including the nurse AIs and RAs, involved in this project.

Members of the nursing stream are included as AIs on this project: AI Moore is a Nurse Practitioner working at QCH and a large mixed suburban ED which frequently transfers patients for paediatric surgical services; AI Mulready is a clinical educator and CNC across the Sunshine Coast HHS which includes not only the large Sunshine Coast University Hospital but also rural and regional centres (Nambour, Gympie).

The RAs that will be funded as part of this project are anticipated to be ED nursing staff; and study champions from the nursing group will also be sought for the project. Further information on the inclusion of nursing staff in this specific project is included in the response to reviewer 1.

Further work into barriers and enablers as part of knowledge translation and implementation will include the multidisciplinary team.

Reviewer 3 Assessment

Strengths:

This is a strong team. This is an existing study that is being co-ordinated at Perth Children's Hospital, and the study has been endorsed by the PREDICT network. The investigator team convincingly have the required skills, expertise and capacity to conduct this research in the required time frame. There also appears to be support from the relevant institutions at which the investigators are based.

Weaknesses:

All (or most) of the investigators are paediatric ED (or surgical) staff. This is fantastic and I'm confident that governance, ethics, recruitment, data collection etc will be successful. But who is conducting the analyses / interpretation and write-up? This is not accounted for in the budget. It is alluded to that this component of the research will be conducted by the central co-ordinating site (Perth Children's Hospital), but then why are these people not included

as investigators in the application? What assurance can the applicants provide that the required epidemiological and statistical expertise is present (or at least accessible) within the team to conduct the analyses?

Comments/Suggestions: Nil

Applicant - Response to Reviewer 3
All study investigators will be actively involved in interpretation of results and the write up process. Opportunities will also exist for secondary papers to be written by other members of the study team (again with active input from the team). The investigator team listed as part of this grant application is focused heavily on the Queensland based part of the team with the two Principal investigators from the central study team listed (Prof Meredith Borland, and Dr Weihao Lee). The study investigator team is wider than this and includes personnel from the WA team and interstate. Statistical and health economics expertise is being provided by the central study team (WA based) – a statistician from the Kids Telethon Institute is involved in the project and further support will be provided by the University of WA. The PREDICT network (with which this project is affiliated) also has access to and established collaborations with relevant expertise including further statistical support if required and health economist and knowledge translation experts.

Review Criterion 4 - Budget (10%)

Definition

The reviewer was asked to assess how well your grant application meets these requirements:

- Appropriateness of expenditure
    - Is the budget appropriate, justified and feasible relative to the research opportunity and research design?
- Impact of expenditure
    - Does this expenditure represent good value for the investment?

Please also refer to Section 5, Guidelines for Budget in the Queensland Research Program Guidelines for eligible and ineligible expenditure items.

(Please note, a revised budget table must be attached should there be any changes to the original submission - the upload facility is available at the end of the document.)

Reviewer 1 Assessment

Strengths:

Requested budget is reasonable and achievable.

Leverages other funding sources for other sites.

Weaknesses:

It is unclear from the proposal what proportion of the study(both participants and manu script/output development) will be contributed from the Queensland sites subject to this application.

Comments/Suggestions:

Suggest clarifying the proportionate contribution to the study that will be achieved by this grant. Is it just participant recruitment? or is it significant contribution to study design and output creation?

Applicant - Response to Reviewer 1
It is estimated that Queensland sites will contribute to at least 30% of total recruitment. This is based on annual paediatric census for the proposed Queensland sites (130,000-135,000 ED paediatric presentations p.a.; QCH alone sees ~ 80,000).

Queensland investigators (Drs Phillips (ED) and Patel (Paediatric General Surgeon)) have been involved in study design, and protocol development and refinement, across the last 2 years. All investigator team members will be involved in results interpretation, outcome creation, and translation of research findings into acute clinical care practice (e.g. through g uidelines/education/other specific measures).

Reviewer 2 Assessment

Strengths:

Appropriate, already funding from other States.

Weaknesses: Nil

Comments/Suggestions: Nil

Applicant - Response to Reviewer 2
Nil specific.

Reviewer 3 Assessment

Strengths:

This is sound, with only minor queries.

Weaknesses:

Why is there only 8 hrs set up at each site? This seems a significant underestimate, and there is a risk of not setting up properly? It may be that I'm misunderstanding what "set up" involves. Can the applicants please expand on what "set up" involves, to demonstrate that it is achievable in 8 hrs? If not, then perhaps increase the allocated time and budget for this bit.

Comments/Suggestions: Nil

Applicant - Response to Reviewer 3
We thank the reviewer for their concerns.

Set time is an estimate only and the time allocated would be spread across a few different time periods. Set up in this instance pertains to specific study RA education and site preparation for study initiation (poster placement, recruitment form and PICF placement, finessing of study flow, study logs etc). It does not include governance preparation (which needs to occur prior to funding agreement completion and will include site specific parent information and consent forms from master study documents) or preparation of site-specific electronic logs and educational materials. Study PIs will take responsibility for governance preparation and initial education of clinical staff; with study RAs continuing this education as required throughout the study period as needed and supported by study PIs.

Review Criterion 5 - Translation into Practice (10%)

Definition

The reviewer was asked to assess how well your grant application meets these requirements:

- Evidence of the practical ability and plan to communicate and disseminate findings into the research and emergency care community
  - Is there evidence of stakeholder engagement to help support the translation of the research outcomes into practice?
- Evidence and plan to translate outcomes into practice
- Benefits to patients and cost savings and/or released capacity to health systems

Reviewer 1 Assessment

Strengths:

Likely to find outcomes that will be readily translatable in to ANZ practice. Team has a track record of achieving this through PREDICT.

Weaknesses:

Extent of potential benefits hard to quantify at this stage.

Comments/Suggestions: Nil

Applicant - Response to Reviewer 1
Many thanks for the review - PREDICT and the study team are committed to ensuring research is relevant, and translatable into emergency practice.

SPASMs aims to streamline care and better identify which children and adolescents need surgery, which are suitable for expectant management at home and which require further investigation and or observation, whilst avoiding unnecessary investigations and delays in care. These benefits are expected to be realised beyond the study sites, and may assist in the decision to transfer patients for definitive care (with clinician, hospital and ambulance resource, and patient benefits). It will also improve our knowledge and understanding about how appendicitis presents and is managed across an Australian context, including variations in care.

Reviewer 2 Assessment

Strengths:

PREDICT has strong track record in this space of translating evidence to practice.

Weaknesses:

Nil mention of addressing facilitators and barriers to use of particular tools, if practice change following validation is the goal, these data should also be collected, from medical, surgical and ED nursing staff (who are often the advocates for/against particular treatment).

Comments/Suggestions: Nil

Applicant - Response to Reviewer 2
The study team agrees that identification of barriers and facilitators is crucial to successful knowledge translation and practice change. While this is beyond the scope of this

particular grant application, it forms a key part of a parallel project being undertaken by the investigator team and Dr Wei Hao Lee as part of his PhD on the care of children with appendicitis and abdominal pain. This qualitative work is intended to involve Queensland participants, across streams and specialties. Understanding variation in practice within and between institutions is also crucial to any knowledge implementation efforts – this will be addressed within this grant application.

Reviewer 3 Assessment

Strengths:

The applicants are well placed to facilitate knowledge translation across a variety of spheres (practice, policy), and have a very well-thought out, applied plan for doing so. This is a strength of the application, and the applicants should be commended.

Weaknesses: Nil

Comments/Suggestions: Nil

Applicant - Response to Reviewer 3
Many thanks for the review.

Overall Comments / Suggestions and Upload Facility

Reviewer Assessment

Reviewer 1: Thank you for this submission. It is well thought through and well written.

Reviewer 2: Addressing an area of need, and a well written proposal with a well designed study.

However, there is no consideration of collection of information to enable translation of findings. This would be very valuable to collect while there are dedicated research staff.

Reviewer 3: The study is already being conducted (and is funded) outside of Queensland, and the proposed funding is to add 3 sites from Queensland, which the applicants propose will improve the applicability of findings of the study to the Queensland context. The full study will allow the researchers to describe the current diagnostic and management approaches in Australian and New Zealand for paediatric abdominal pain and appendicitis. To gain the full benefit of the funds, I think there is merit to the researchers spending some time thinking specifically about how the addition of the Qld sites will benefit the study over and above the existing funding, and also how the results may specifically apply to Queensland. This would require some sub-analyses specific to Queensland. Overall, the researchers present a compelling argument for a soundly designed research proposal. This application complements an existing (currently funded) work, so is low-risk in many respects. It is well-reasoned, and has high potential for translation into practice and impact on ED.

Applicant - Response
We thank all reviewers for their time, the highly valuable feedback provided and the opportunity to address the concerns and queries raised, including Queensland specific sub-analyses and further strategies to enhance translation of findings (provided in previous section responses). The further suggestions/considerations provided to enhance the project have been much appreciated.

Applicant - Upload Facility

Please use the upload facility to provide any documentation required to support your response to review.
No files have been uploaded
(For example, revised budget, additional investigators etc.)
